# Supplementary material for: Dynalign II: common secondary structure prediction for RNA homologs with domain insertions
Source: Nucleic Acids Res. 2014 Nov 21;42(22):13939–48. doi: 10.1093/nar/gku1172 (PMC4267632; doi:10.1093/nar/gku1172)
Supplement: SUPPLEMENTARY DATA [file supp_gku1172_nar-02021-z-2014-File012.zip › manual/Overview/Installation_Instructions_Windows.html]

RNAstructure Installation and Overview: Installation Instructions for Windows Systems


|  |  |  |
| --- | --- | --- |
|  | RNAstructure Installation and Overview Installation Instructions for Windows Systems | - Contents - Index |
| This page only gives details on how to install the package. For information on how to build the package, please refer to the Building Instructions and Building Requirements pages. To install the graphical user interface (GUI), follow these steps:   1. Download the Windows GUI from the Mathews Lab RNAstructure page. See the note below if you are not sure about whether to use 32-bit or 64-bit. 2. Double-click the zipped package to view the contents. 3. Double-click setup.exe and use the Windows installer to install the package. 4. RNAstructure can now be started from the Start menu. 5. The complete manual for the GUI can be found here. It can also be found under the help menu item within the program.   Special note about Example Files:  The example files that come with RNAstructure will be installed in your user data folder in a new folder called RNAstructure\_examples. This is "My Documents" on Windows XP and "Documents" on Windows 7 and Windows 8.  To install the text interfaces, follow these steps:   1. Download the text interfaces for Windows from the Mathews Lab RNAstructure page. See the note below if you are not sure about whether to use 32-bit or 64-bit. 2. Extract the files from the zipped package by double-clicking and dragging the contents to the desired location. 3. Specify the DATAPATH environment variable as explained here. 4. The executables are in the exe folder. 5. Note that the -smp versions of programs (for parallel processing) also rely on libiomp5.dll, which is provided in the exe directory. It needs to be in the same directory a the -smp exe. 6. The complete manual for using the text interfaces is available here.   A Note about 32-bit and 64-bit:  The 32-bit verions will work on either 32-bit or 64-bit versions of Windows. The 64-bit will only work on 64-bit versions of Windows. The 64-bit version of RNAstructure can address more memory, and hence longer sequences can be analyzed using the 64-bit version. For the GUI, the 64-bit installer will stop if you have a 32-bit version of Windows.  If you are not sure which version to download because you do not know what version of Windows you have, the 32-bit is a safer bet.  Windows 7 Compatibility:  RNAstructure is Windows 7 compatible. Prior versions of RNAstructure required the user to run in "Windows XP Compatibility Mode," but that requirement is removed.  Running on Windows 8:  RNAstructure is now Windows 8 compatible. An update was uploaded on 5/16/2013 to make the version 5.5 compatible. | | |
| Visit The Mathews Lab RNAstructure Page for updates and latest information. | | |
